# Dynamically Switchable Polarization-Independent Triple-Band Perfect Metamaterial Absorber Using a Phase-Change Material in the Mid-Infrared (MIR) Region

**DOI:** 10.3390/mi12050548

**Published:** 2021-05-11

**Authors:** Dongdong Xu, Fenping Cui, Gaige Zheng

**Affiliations:** 1 Jiangsu Key Laboratory for Optoelectronic Detection of Atmosphere and Ocean, School of Physics and Optoelectronic Engineering, Nanjing University of Information Science & Technology, Nanjing 210044, China; 20191217011@nuist.edu.cn (D.X.); cuifenping@nuist.edu.cn (F.C.); 2 Jiangsu Collaborative Innovation Center on Atmospheric Environment and Equipment Technology (CICAEET), Nanjing University of Information Science & Technology, Nanjing 210044, China

**Keywords:** germanium-antimony-tellurium, triple-band absorber, mid-infrared regime, near unity absorption

## Abstract

A tunable metamaterial absorber (MMA) by reversible phase transitions in a mid-infrared regime is theoretically investigated. The absorber is composed of a molybdenum (Mo)-germanium-antimony-tellurium (Ge_2_Sb_2_Te_5_, GST)-Mo nanodisk structure superimposed on the GST-Al_2_O_3_ (aluminum oxide)-Mo film. Studies have shown that the combination of the inlaid metal-medium dielectric waveguide mode and the resonant cavity mode and the excitation of the propagating surface plasmon mode are the main reasons for the formation of the triple-band high absorption. Additionally, through the reversible phase change, the transition from high absorption to high reflection in the mid-infrared region is realized. The symmetry of the absorber eliminates the polarization dependence, and the near unity absorption efficiency can be maintained by incidence angles up to 60°. The presented method will enhance the functionality of the absorber and has the potential for the applications that require active control over light absorption.

## 1. Introduction

Thanks to Padilla et al., the perfect metamaterial absorber (PMA) was first proposed in 2008 in the field of electromagnetic waves, and thus PMAs has been developed rapidly [1]. In recent years, there has been a great need for perfect absorbers (PAs) in stealth [2,3], photovoltaics technology, sensors [4,5], and other opto-electronic devices [6,7]. High-resolution manufacturing technologies for nanostructures are becoming more common as they are updated, and PMAs are receiving more and more attention due to their multiple light control functions. PMAs have been extensively studied for the novel electromagnetic (EM) phenomena and applied in various aspects, such as selective modulation, nano-lenses, bolometers, thermal emitters, Q-switches for infrared lasers, and many other areas [8,9,10,11,12,13,14,15]. Among the above studies, PMA platforms have been designed and manufactured in the spectral ranges of visible [16,17], infrared [18,19], and terahertz [20,21].

The key to highly absorbing media is the design of resonant structures that take advantage of the inherent loss of metal, such as resonators [22,23,24], nanowire arrays [25], and nanodisk arrays [26], which can be driven by both the electrical and magnetic fields of the radiation, as well as the perfect or optimal impedance matching of the metamaterial to the vacuum, which leads to resonant absorption of the radiation. However, it is well known that the resonant frequency is affected by the structural parameters of the structure, but once a micro-nano structure is designed and manufactured, its geometry and size are fixed, and thus it is difficult to tune the resonance. Then the resonant response can only be adjusted by changing the dielectric properties of the external medium. Thus far, many new studies have yielded exciting results, such as graphene [27,28], phase-change materials (PCM) [29], and transparent conducting oxides [30,31]. Ge_2_Sb_2_Te_5_ (GST) is a phase-change material in which an increase in temperature within the material under the direct action of an external electric field or thermal stimulus leads to a transformation of the internal structure from the amorphous (AGST) state to the crystalline (CGST) state, accompanied by a series of changes in the physical properties of the material, such as the refractive index and electrical conductivity [32,33]. Notably, by heating GST to its melting point and rapidly cooling it to room temperature, it is possible to make the GST revert from the crystalline to the amorphous state, with a switching rate capable of reaching megahertz.

In this work, a nanodisk hexagonal array design triple-band absorber is proposed, where the absorber consists of a Mo-GST-Mo nanodisk structure superimposed on a GST-Al_2_O_3_-Mo film. The GST film is proposed to tune the absorption by phase transition. The formation of three resonant absorption peaks in the MIR range of 8–20 μm can be observed in the crystalline phase. The numerical values show that the absorption peaks are at λ_1_ = 11 μm, λ_2_ = 15.2 μm, and λ_3_ = 17.6 μm with 99.3%, 99.9%, and 96.5% absorption, respectively. Additionally, the transition of the device from high absorption to high reflection in the amorphous phase makes this design more attractive. The absorber has good triple frequency absorption performance, good operating angle polarization tolerance, and dynamic tunability advantages, which may be beneficial for a variety of MIR applications.

## 2. Model and Methods

**Figure 1:**
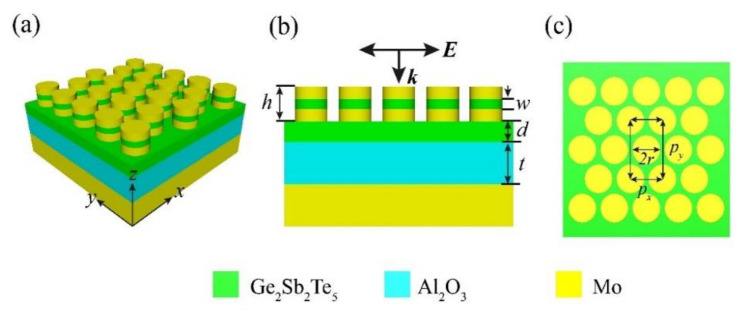
(**a**) Schematic diagram and (**b**) a cross-sectional view of the Mo-GST-Mo nanodisk arrays. (**c**) A schematic top view of the entire structure. The overall height of the Mo-GST-Mo nanodisk is *h*, the thickness of the GST interspersed in the middle is *w*, and the heights of the underlying GST film and Al_2_O_3_ layer are *d* and *t*, respectively. *r* represents the radius of the nanodisk array. Separately marked are the periods of the arrays in the x and y directions as *p_x_* and *p_y_*_._

The Mo-GST-Mo nanodisk array was superimposed on a Mo substrate separated by GST and Al_2_O_3_ dielectric layers, as shown in sections of the x-z plane in Figure 1a,b. The Mo substrate on the backside of the nanodisk array is used as a reflecting mirror to prevent the transmission of incident light. When the GST is in the crystalline state, the Mo-GST-Mo nanodisk array itself can be regarded as a small weak Fabry—Perot (FP) cavity, and a Fabry–Perot (FP) cavity can be formed between the entire Mo-GST-Mo array and the base Mo substrate. The overall height of the Mo-GST-Mo nanodisk is *h*, the thickness of the interposed GST is *w*, and the heights of the GST film and Al_2_O_3_ layer below are *d* and *t*, respectively. As shown in Figure 1c, the radius of a single Mo-GST-Mo cylinder is *r*, and the individual periods along the x and y directions are labeled *p_x_* and *p_y_*, where $p_{y}=\sqrt{3}p_{x}$. The interaction between light and the hybrid structure was studied by the three-dimensional finite difference time domain (3D-FDTD) method. Periodic boundary conditions were used along both the x and y directions, respectively, and perfectly matched layers (PML) were set at both the top and bottom boundaries. A plane wave light source was used to incident positively on the array from the z-direction, while the plane wave electric vectors were all parallel to the x-direction by default.

In this design, GST exhibits two stable structural phases, i.e., the amorphous (AGST) and the crystalline (CGST) states, and can switch rapidly between the two under external electric or thermal stimulation, while the lattice state can be stably maintained. The dielectric constants of the two stable structural phases of the GST films were taken from the literature [34]. The dielectric constant of Al_2_O_3_ was taken from Palik’s optical constants handbook [35]. The dielectric constant of Mo in the infrared is modeled by the Drude model:

(1) $$\varepsilon =1-\frac{\omega_{p}^{2}}{\omega \left(\omega +i\omega_{c}\right)}\;$$

where *ω* is the incident plane wave, the plasma frequency is *ω_p_* = 2π × 1.81 × 10^15^ rad^−1^, and the damping constant is *ω_c_* = 1.24 × 10^13^ s^−1^ [36].

The reflection of electromagnetic waves (R) is calculated by setting the monitor at the top, and since the Mo substrate at the bottom is thick enough to act as a reflector to completely block the transmission of incident light to obtain maximum absorption, the absorption (A) can be defined as A=1−R−T=1−R. 

## 3. Results and Discussion

One of the most encouraging aspects of metamaterials is that the desired optical properties are achieved by configuring structures that go beyond the inherent properties of the material included. Mo is used as a retro-reflector to avoid incident light passing through the metal film, thus reducing the fugitive work rate and thus increasing the absorbance to close to unity. For GST films, the dielectric constant changes dramatically once the phase change is triggered by a thermal field. The geometrical parameters were initially set to *t* = 100 nm, *d* = 160 nm, *h* = 350 nm, *w* = 150 nm, *r* = 460 nm, and *p_x_* = 1000 nm. We will use them throughout the article unless otherwise stated.

**Figure 2:**
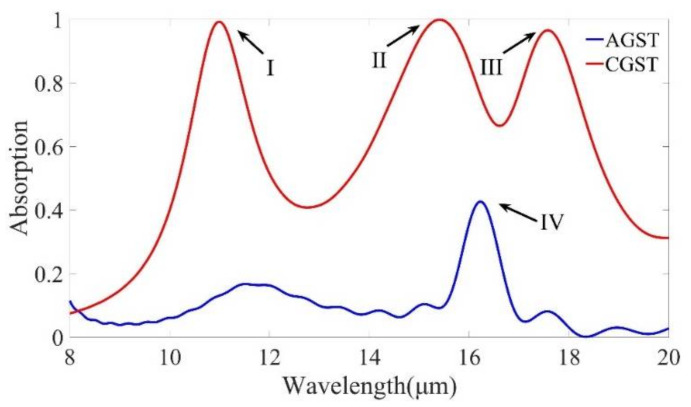
Total absorption at normal incidence for a structure with amorphous (AGST) and crystalline (CGST) stable structural phases.

Figure 2 shows the absorption spectra of the composite structures in the AGST (blue) and CGST (red) cases. The nanodisk structure can excite three absorption peaks in the GST crystalline phase state in the mid-infrared range, which are marked as resonance mode (I) (absorption > 99.3%), resonance mode (II) (absorption > 99.9%), and resonance mode (III) (absorption > 96.5%), and the corresponding wavelengths are 11, 15.2, and 17.6 μm, respectively. Under amorphous conditions, the absorption peak at resonance is located at 16.2 μm, which is marked as resonance mode (IV) (absorption is about 42%). Surprisingly, as the GST transitions from amorphous to a crystalline stable structure phase, the device transitions from full absorption to high reflectivity. Due to the reversible phase change, significant wavelength shift and high absorption contrast can be achieved.

**Figure 3:**
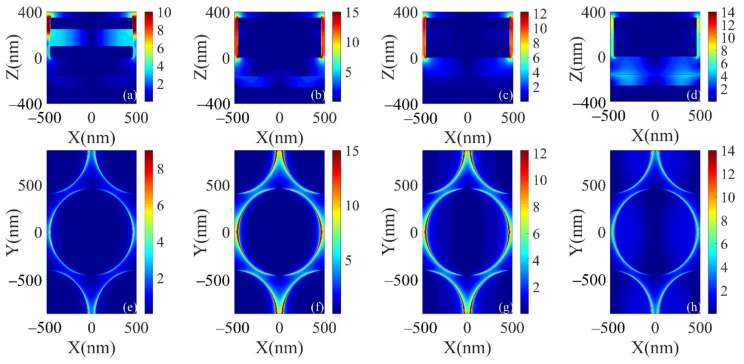
(**a**–**d**) Electric field distribution curves of resonance modes (I), (II), (III), and (IV) in the x-z plane at resonance wavelengths of 11, 15.2, 17.6, and 16.2 μm, respectively. (**e**–**h**) Electric field distribution curves of resonance modes (I), (II), (III), and (IV) in the x-y plane at resonance wavelengths of 11, 15.2, 17.6, and 16.2 μm, respectively.

In order to reveal the physical origin of near unity absorption of MMA, the electric field was studied quantitatively. In general, two different resonance modes can be observed from Figure 3. In resonance mode (I), the electric field is mainly concentrated on the side of the upper part of the nanodisk. In contrast, in resonance modes (II) and (III), the electric field is confined to the sides of the entire Mo-GST-Mo nanodisk, and the electric field distribution suggests that this resonance mode has excited localized surface plasmon excitonic resonance (LSPR) that enhances electromagnetic (EM) waves around the nanodisk array and dominates the absorption. Due to the negative dielectric constant of the metal Mo, LSPs with a field enhancement effect will be excited at the metal–dielectric interface. According to the theory of single-waveguide cavity [37], under incident light, only modes that meet the phase matching condition may exist in the air cavity. The specific formula is as follows:

(2) $$\left|2Lk_{LSP}\left(\omega \right)+\Delta \varphi_{1}+\Delta \varphi_{2}\right|=2\pi m$$

where *L* is the length of the cavity, $k_{LSP}\left(\omega \right)$ is the wave vector of the plasmon at frequency ω, $\Delta \varphi_{1,2}$ is the phase shifts of plasmon reflection at the upper and bottom of the cavity, and m is the mode number (different measured resonance symbols). Certain specific wavelengths ((I), (II), and (III)) of incident light satisfy Equation (2). From Figure 3b,c,f,g, it can be seen that some energy penetrates through the nanodisk into the lower GST film and Al_2_O_3_ layer. When the incident light penetrates the upper metal, the lower metal acts as a mirror, which can reflect the surface plasmonic excitations at the lower metal-dielectric boundary, and the surface plasmonic excitations between the upper metal-dielectric interface and the lower metal-dielectric interface will undergo phase extinction interference. The resonant cavity mode is excited, resulting in strong absorption of light. This resonant cavity mode and the local surface plasmon excitations resonate leading to the strong absorption of resonant modes (I), (II), and (III). As for resonance mode (IV), the incident electromagnetic wave excites resonance between the entire nanodisk structure and between the GST film and Al_2_O_3_ layer, and the strong coupling over a large area may lead to energy.

**Figure 4:**
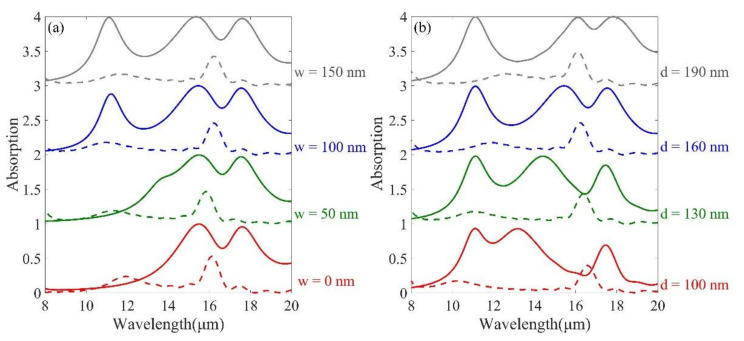
(**a**) Absorption spectra of the middle GST layer of nanodisks with crystalline and amorphous phases at different heights. (**b**) Absorption patterns of bottom GST films with crystalline and amorphous phases at different heights. The other geometric parameters are the same as in Figure 2.

To further confirm the relationship between the physical mechanism and structure of the high absorption of MMA, we quantitatively analyzed the specific geometric parameters of the structure. Firstly, the effect of GST layer thickness on the absorption spectrum was analyzed. For GST in the crystalline phase, it can be seen from Figure 4a that there are only two absorption peaks when w is 0. As w increases, the short wavelength gradually splits another absorption peak, at which time the combination of Mo and GST in the nanodisk forms a dielectric waveguide, leading to the appearance of resonance mode (I), but has almost no effect on resonance modes (II) and (III). From Figure 4b, it can be seen that the increase of GST film thickness d leads to a large red shift of resonance mode (II) and the enhancement of resonance intensity of resonance mode (III). From Figure 4a,b, it can be seen that for GST in amorphous phase, there is almost no change in resonance mode (IV).

**Figure 5:**
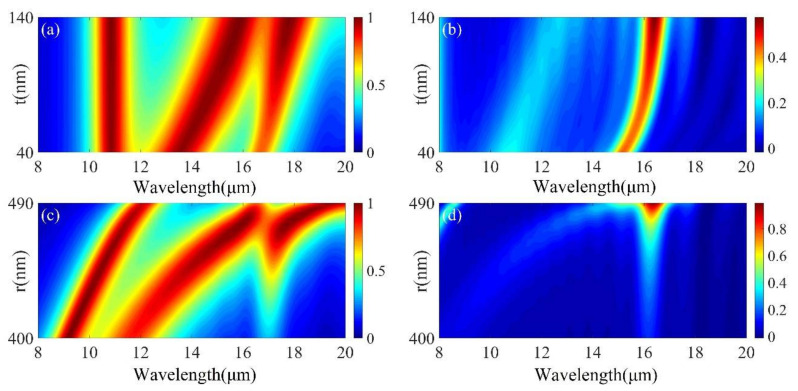
(**a**,**b**) Absorption patterns of Al_2_O_3_ layers with crystalline and amorphous phases at different heights. (**c**,**d**) Absorption patterns of various radiuses of Mo arrays with crystalline and amorphous phases, respectively. The other geometric parameters are the same as in Figure 2.

With a fixed lattice period, the thickness of the Al_2_O_3_ layer will affect the field intensity and Q factor (which is defined by *λ_res_*/Δ*λ*, where *λ_res_* is the center wavelength of the peak) of the resonant mode (III). As shown in Figure 5a,b, the simulation results show that both resonance modes (I) and (II) can maintain more than 95% of the peak absorption when other parameters are fixed and *t* is increased from 50 to 200 nm. However, the spectral line of resonance mode (III) is red-shifted and gradually increases in intensity with the increase of *t*, which can improve the absorption efficiency of the structure. 

Each Mo-GST-Mo cylindrical array and the GST-Al_2_O_3_-MO layer below acts as an FP resonator, and the peak wavelength of the FP mode is approximately: *λ_FP_* = 2*tn_eff_*, where *t* is the depth of the cavity and *n_eff_* is the effective refractive index of the cavity. When *r* increases, due to the increase in the effective resonant wavelength, the resonant mode will be red-shifted. As shown in Figure 5c,d, when other parameters are fixed, when *r* changes from 400 to 490 nm, the three absorption peaks are red-shifted, and the resonance mode (II), (III) maximum absorption gradually increases. Meanwhile, the resonance mode (IV) in the non-crystalline state also increases rapidly with the increase of *r*, which affects the contrast of the absorber, so for the radius *r*, it is more suitable to choose 460 nm.

**Figure 6:**
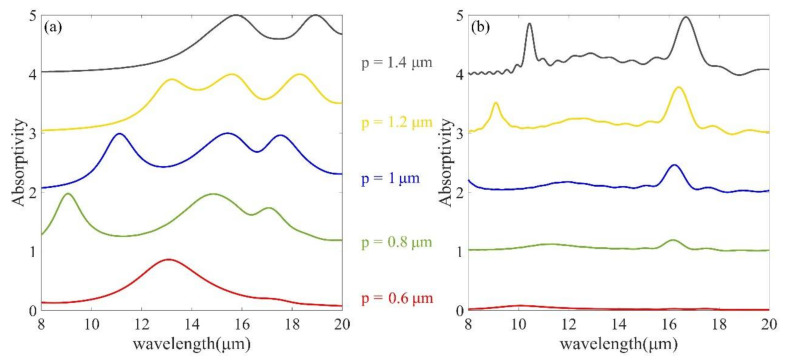
(**a**,**b**) Absorption spectra of nanodisk arrays with crystalline and amorphous phases at different periods, respectively. The other geometric parameters are the same as in Figure 2.

It can be interestingly found in Figure 6 that when *p* is only 0.6 μm, the crystalline phase nanodisk array has only one resonance peak and the amorphous phase has almost zero absorption, allowing for ultra-high contrast. As the period increases, the crystalline phase spectrum appears as three absorption peaks and red-shifts, while the amorphous phase resonance intensity gradually increases. Thus, we can control different resonance absorption ranges by adjusting the period of MMA.

**Figure 7:**
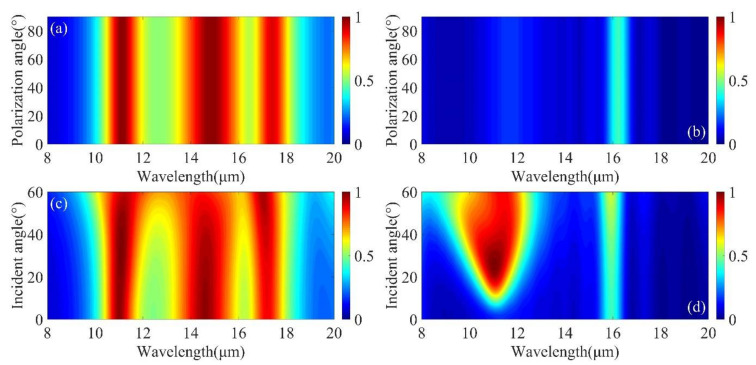
(**a**,**b**) Spectral response of nanodisk arrays with crystalline and amorphous phases at different polarization angles (0–90°). (**c**,**d**) Simulated absorption spectra of Mo arrays with crystalline and amorphous phases at different (0–60°) incidence angles, respectively. Other geometric parameters are fixed and are the same as used in Figure 2.

**Figure 8:**
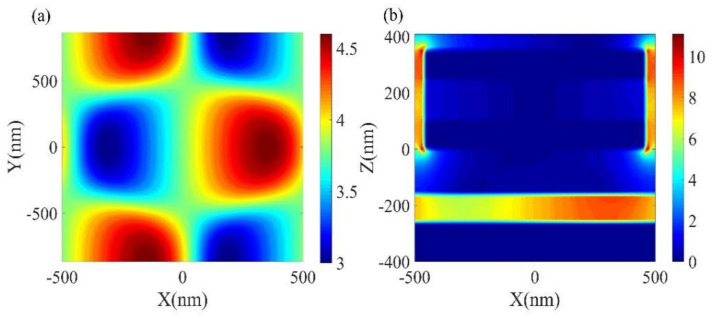
(**a**) Electric field distribution in the x-y plane with a resonance peak of 11 μm at an incident angle of 30°. (**b**) Electric field distribution in the x-z plane with a resonance peak of 11 μm at an incident angle of 30°.

Finally, the absorption stability of the structure was investigated under changes in polarization and incidence angle. One advantage of the hexagonal structure is azimuthal symmetry, while the hexagonal array has three-fold rotational symmetry, which makes the absorption insensitive to the polarization angle at normal incidence [38,39,40]. At perpendicular incidence, the polarization angle is in the range of 0° to 90°, with only 0° for the electrodes in the x-direction and 90° for the y-direction. It can be clearly seen that there is no change in absorbance as shown in Figure 7a,b. Figure 7c,d shows the simulated absorption of the proposed structures at 0–60° incidence angles. In the crystalline phase, high absorption is still achieved even when the incidence angle reaches 60°, but in the amorphous phase, when the incident light is tilted at a certain angle, an additional absorption peak appears in the absorption spectrum. Thus, as shown in Figure 8a,b, we calculated the x-y and x-z plane electric field at the resonance wavelength at 30° incidence angle and found that an electric field enhancement appears in the GST layer and that the coupling between the LSP of the disks and the cavity modes in the GST layer forms a new absorption peak.

## 4. Conclusions

In summary, a triple frequency PMA with reversible phase transition has been proposed and employed. It allows perfect absorption in the mid-infrared region at three frequencies. By using the field concentration properties of the FP cavity to match the tunable optical properties of the GST, it can excite localized plasmon resonances and combine them with dielectric waveguide modes to convert high absorption to high reflectivity. It is expected that this work will find applications in areas where radiation modulation or active control of light absorption is required.
